# Circumventing Heisenberg: non-invasive methods for cell biology

**DOI:** 10.1007/s00709-023-01884-0

**Published:** 2023-07-26

**Authors:** Peter Nick

**Affiliations:** Joseph Gottlieb Kölreuter Institute Für Plant Sciences, Karlsruhe Institute of Technology, Karlsruhe, Germany

Scientists tend to think that their work is “objective.” While the majority would agree that this claim is certainly not true for the interpretation of data, because this interpretation depends on pre-formed notions called working hypotheses, still most would claim that the data themselves are “objective” in the sense that they are independent from the person, who collected them. However, even this aspect of scientific objectivity needs to be questioned. The reason for this skepticism is not less mistrust of moral integrity, but the limitations imposed by methodology. We can only see what our tools allow us to see. As trivial as this statement may appear, it has been shaping scientific concepts. For physics, the principal methodological constraints imposed on human knowledge have been discussed extensively. In his famous uncertainty relation, Heisenberg (1927) even quantified these constraints caused by the fact that our method of observation will interfere with the observed object, such that we cannot determine its location and its dynamics at the same time. The methodological breakthroughs of the last decades have not only advanced biology, but also have inserted numerous layers of technology between the observer and the object of observation. In other words, our perception has turned progressively indirect. As long as we are aware of this, we can at least safeguard “objectivity” by appropriate controls. However, at the very moment when we start to naively believe what we see, we are prone to be fooled by methodological artifacts. This problem is even accentuated by the fact that, for many approaches, we have to modify our object to render it accessible to our methodology. For instance, to see the subcellular localization of a protein of interest using GFP technology, we need to generate fusions and transform the organism under scrutiny. This will not only change the protein, but also the organism. While we will never escape the limitations imposed by the fact that our observation is always filtered by the accessible methodology, we might at least try to resort to methods that are non-invasive and leave the object of our observations as untouched as possible. Two contributions to the current issue address such non-invasive technology.

The contribution by Cota-Sánchez et al. (2023) is using synchrotron radiation micro-computed tomography to collect morphological details from the hidden inside of intact, developing flowers of the prickly pear, *Opuntia polyacantha*. While it is possible to address such anatomical details by conventional microtomy physically cutting the object into slices, it is cumbersome and also partially ambiguous to reconstruct the original three-dimensional structure from such sections. This gets particularly difficult in delicate structures that are easily distorted during embedding and sectioning. Synchrotron-based approaches allow to leave the object intact and have been especially valuable in structures that are otherwise recalcitrant to microscopical inspection, such as root nodules in legumes (Rivard et al. 2019). Synchrotron beams are very bright, strongly polarized, can be tuned with respect to spectrum and timing, and, thus, have been an efficient tool to extract spatial information from the inside of compact, otherwise opaque objects. The integration of powerful computing allows to increase the spatial resolution to an extent that even cellular details can be discerned. In the current study, the authors ask for the developmental origin of staminodia, sterile stamina. Using segmentation strategies, they are able to reveal even tiny details of vascular networks, such as mucilage, the structure of the epidermis, and the three-dimensional setup of the mesophyll. They can show that there is a continuous transition between tepals (floral leaf organs found in basal Angiosperms that only later in evolution differentiate into sepals and petals) and true stamina. Based on the structural details revealed by their non-invasive methodology, they can show that the staminodia are derived from tepals. This finding bears on concepts of floral development—these have been shaped by the ABC model, proposing how four whorls of floral organs are determined by combinations of transcription factors encoded by the ABC genes (for an updated version of this model see Theißen et al. 2016). However, this model has been developed in highly derived model organisms, such as *Arabidopsis thaliana* or *Antirrhinum majus*. In basal angiosperms, there are no strict borders between floral organs, but rather smooth transitions. For instance, in *Clematis*, belonging to the basal Ranunculaceae, four types of stamina could be discerned that represent different positions in the transition from a sterile tepal to a fully fertile stamen (Li et al. 2021). This cannot easily be accommodated into the ABC model but rather speaks for the so-called fading border concept (for review see Chanderbali et al. 2016). This concept proposes gradients of transcription factors, whose relative activity defines organ shape in a continuous gradient, and not in the form of a qualitative decision. The findings made possible by the synchrotron are more in line with this fading border model. Thus, a new technology not only allows insights into otherwise hidden parts of an object, but leads to new concepts.

Climate change imposes tremendous challenges upon agriculture, especially in tropical and subtropical countries. To cope with this challenge, new crop plants with elevated stress resilience are mandatory. This simple conclusion has initiated tremendous breeding activity. However, to phenotype, the stress status of a plant is far from trivial. When stress damage becomes manifest, it is usually too late already. Thus, a new methodology is needed to monitor plant stress. However, many of the methods used for this purpose are destructive, meaning that the entire plant or at least parts of it have to be sacrificed, such that it is very difficult to follow stress adaptation over time. Therefore, non-invasive approaches are crucial. This is, in a nutshell, the mission of the contribution by Singh (2023). Using Ashwagandha (*Withania somnifera*), a medicinal plant belonging to the Solanaceae and central to traditional Ayurvedic medicine as a paradigm, this author develops and validates a couple of stress readouts that are non-invasive. The study is based on an LI-COR spectroradiometer, which can generate a plethora of readouts that might allow to infer the stress status but remain meaningless if they are not grounded on physiology. This is exactly what the authors do, comparing prolonged drought stress of different severity as compared to non-stressed controls. To map the stress status of the plants, they first start with a large number of typical stress readouts, such as leaf relative water content, osmotic potential of the leaves, pigment content, cellular integrity by electrolyte leakage, root hydraulic conductance, carbon isotope ratios (an integrative reporter for stomatal aperture), and even the content of the value-giving metabolite, the withanolides. All these readouts are destructive, though. Now, they search for non-invasive readouts that can be measured with the spectroradiometer and project those to the stress output assessed by the conventional parameters. For instance, they can show that the photosynthetic reflectance index correlates with the relative water content of a leaf. By measuring specific wavelengths in the red and near-infrared range, they can assess chlorophyll content, chlorophyll a/b ratio, and the activity of photosystem I, while fluorescence can be used to address photosystem II. Furthermore, non-photochemical quenching can be used to report the status of the xanthine cycle. The author not only describes these non-invasive parameters, but also comes up with physiological explanations for their changes under drought stress. The study is interesting beyond the model, *Withania somnifera*, because it demonstrates which of the numerous data that can be collected with a spectroradiometer are informative in the context of drought stress.

Both contributions use non-invasive technology to extract knowledge on the inner state of organisms that otherwise would be difficult to acquire. It is not only new technology, though. The interpretation of the synchrotron images requires a thorough conceptual understanding of flower morphogenesis. The interpretation of the non-invasive stress readouts requires calibrating experiments in the same system along with an elaborate model of stress metabolism. Thus, in both cases, the non-invasive technology is accompanied by hypothetical models of the respective phenomenon. We may not be able to observe things as they are, because they will always be changed, at least to a certain extent, by our observation. However, we are able to infer things how they are.
